# Named entity recognition for Chinese electronic medical records by integrating knowledge graph and ClinicalBERT

**DOI:** 10.3389/frai.2025.1634774

**Published:** 2025-09-10

**Authors:** Xiang Xu, Zhengxiong Li, Hongwei Zhang, Kai Ma

**Affiliations:** School of Medical Information and Engineering, Xuzhou Medical University, Xuzhou, China

**Keywords:** named entity recognition, ClinicalBERT, Chinese electronic medical records, knowledge graphs, BiLSTM, CRF

## Abstract

**Introduction:**

General purpose language models often struggle with accurately identifying domain specific terminology in the medical field, resulting in suboptimal performance in named entity recognition (NER) tasks. This challenge is particularly pronounced in Chinese electronic medical records (EMRs), which lack clear word boundaries and contain complex medical expressions.

**Methods:**

This study proposes a novel NER method for Chinese EMRs that integrates ClinicalBERT, a language model pre trained on clinical corpora, with structured knowledge from a medical knowledge graph. Entity representations derived via Translating Embeddings (TransE) are incorporated to inject external semantic knowledge. Furthermore, the model fuses multiple character level features, including positional labels, contextual category clues, and semantic embeddings, to enhance boundary detection. The input text is annotated using the BIOES (Begin, Inside, Outside, End, Single) tagging scheme and subsequently encoded by ClinicalBERT. The encoded features are then passed through a bidirectional long short term memory (BiLSTM) network and a conditional random field (CRF) layer for final label prediction.

**Results:**

Experiments conducted on publicly available datasets demonstrate that the proposed approach achieves an *F*1 score of 89.44 percent, surpassing multiple existing baseline models in performance.

**Discussion:**

These findings confirm that the integration of domain specific language modeling, structured medical knowledge, and enriched character level features significantly enhances NER accuracy in Chinese EMRs. The proposed method shows strong potential for practical deployment in clinical information extraction systems.

## 1 Introduction

With the advent of the artificial intelligence era, the use of emerging data technologies in the construction of electronic medical information has been strongly supported by national policies. The modern medical field has introduced intelligent diagnostic systems to assist doctors in disease diagnosis and drug recommendation, these systems usually rely on a large number of patients’ electronic medical records, which serve as important carriers of modern medical diagnosis and contain a large amount of unstructured text data, such as the patient’s medical history records, examination results, medical advice and treatment records, medical records, discharge summaries, and nursing care records (Shan et al., 2024). However, these raw medical data cannot be directly used by intelligent diagnostic systems due to their highly unstructured format and specialized content.

Named Entity Recognition (NER), as one of the key sub-tasks of Natural Language Processing, accurately identifies specific named entities in unstructured text and categorizes them into corresponding entity types (Liu et al., 2022). In the medical field, NER is able to accurately annotate medical domain-specific medical entities including diseases and diagnoses, surgical procedures, anatomical locations, and drug names. The entities extracted through medical NER serve as foundational elements for a wide range of downstream clinical applications, effectively bridging unstructured narrative text and structured decision-making systems. Specifically, high-quality NER enables the automatic construction of structured electronic health records (EHRs), facilitates population of clinical knowledge graphs, and supports rule-based or machine-learning-driven clinical decision support systems (CDSS). For example, accurate recognition of disease names and medication entities can aid in drug-disease interaction detection and pharmacovigilance. Extraction of anatomical locations and surgical terms contributes to precise treatment planning and retrospective clinical auditing. Moreover, NER output can be directly leveraged in downstream tasks such as relation extraction, medical question answering, adverse event monitoring, and cohort identification for clinical trials. In essence, medical NER not only improves data usability but also plays a pivotal role in enabling secondary use of EMRs for intelligent analytics and evidence-based healthcare. Therefore, enhancing the accuracy, robustness, and domain adaptability of NER systems is of paramount importance for the effective deployment of AI in clinical environments. Therefore, numerous NER methods and techniques have been proposed to effectively integrate medical data and extract core information for medical diagnosis. However, due to the complexity and diversity of medical terminology, expert knowledge is required for data annotation, resulting in a serious scarcity of annotated medical data.

With the continuous advancement of deep learning, models such as Convolutional Neural Networks (CNN) (Li et al., 2021) and Bi-directional Long Short-Term Memory (BiLSTM) (Zhou et al., 2016) have been widely used in English NER applications. However, due to structural differences between English and Chinese texts, Chinese named entity recognition still faces significant challenges compared to its English counterpart. For example, words in English are separated by spaces, while Chinese has no explicit word boundaries. In addition, proper nouns in English, such as names of people and places, are often distinguished by initial capital letters, whereas Chinese does not provide such typographic cues. Therefore, recognizing named entities in Chinese requires more refined algorithmic design and the incorporation of domain-specific knowledge into the models.

To distinguish our work from existing knowledge-enhanced NER frameworks, we highlight the key methodological innovations introduced in this study:

First, unlike prior BERT-based knowledge-enhanced NER frameworks that typically perform token-level entity injection or rely on external gazetteers, our model integrates pre-trained knowledge graph embeddings at the character level in Chinese EMRs, which lack explicit word boundaries. This fine-grained fusion allows for more precise disambiguation and boundary localization, particularly for nested or semantically ambiguous entities.

Second, we introduce a multi-source character-level encoding scheme, which incorporates not only contextual representations from ClinicalBERT and entity embeddings from the knowledge graph, but also auxiliary linguistic features such as BIOES (Begin, Inside, Outside, End, Single) tags and contextual categories. This holistic representation captures both structural and semantic cues, which, to the best of our knowledge, has not been jointly explored in Chinese clinical NER.

Third, we design a joint embedding and contextualization mechanism, in which external TransE (Translating Embeddings for Modeling Multi-relational Data) vectors are linearly projected and fused with BERT outputs prior to sequence modeling. Unlike attention-based knowledge injection or post-hoc entity fusion, this early fusion strategy allows for end-to-end joint optimization of internal and external representations.

Finally, while prior studies have primarily focused on general-domain or biomedical corpora in English, our work is tailored to real-world Chinese EMRs, which are noisier, more abbreviation-heavy, and lack high-quality annotations. This necessitates a novel integration strategy that is robust to linguistic irregularities and domain-specific ambiguities.

## 2 Related work

Medical Named Entity Recognition aims to identify and extract key medical entities—such as diseases, drugs, organs, symptoms, and examination results—from medical texts. It serves as a fundamental step for applications such as medical information extraction and knowledge graph construction and is typically regarded as a sequence annotation task. Early sequence annotation primarily relied on rule-based and dictionary-based approaches, which used manually designed rules and feature templates to identify specific types of entities. Although manually crafted rules and dictionaries can be tailored to specific domains and often yield better recognition results, these approaches heavily rely on manual effort and suffer from several limitations, including high labor costs, inefficiency, and poor generalization. With technological advancement, traditional machine learning methods represented by Conditional Random Fields (CRF) (Quattoni et al., 2004) have been increasingly adopted, effectively reducing labor costs compared to rule-based and lexicon-based approaches, but they still suffer from limited semantic understanding, inefficient feature extraction, and slow training. In recent years, artificial intelligence and deep learning technologies have achieved significant success in the field of natural language processing. Compared to traditional machine learning methods, deep learning approaches can automatically learn feature representations, better capture complex semantic features, and model long-distance textual dependencies, thereby improving training efficiency. Currently, mainstream NER approaches utilize Transformer-based deep learning models—such as BERT (Bidirectional Encoder Representations from Transformers), RoBERTa (Robustly Optimized BERT Approach), and ERNIE (Enhanced Representation through Knowledge Integration)—for feature extraction. Feng et al. (2023) proposed a BERT-based NER method for Chinese electronic medical records, which incorporates Char-CNN to learn character-level features and integrates them into vectors generated by the BERT pre-trained model to obtain word representations, which achieved an *F*1 value of 91.64% on the CCKS17 dataset; Wu et al. (2021) constructed a deep learning model by combining head features with the Ra-RC model, which uses RoBERTa to learn medical-specific features and form feature vectors, then applies BiLSTM to capture internal feature correlations, and finally uses CRF to obtain the optimal label sequence. Experiments show that the model achieves *F*1 scores of 93.26 and 82.87% on the CCKS2017 and CCKS2019 datasets, respectively; Wang et al. (2020) proposed an ERNIE-based joint model, ERNIE-joint, designed for both NER and text classification tasks through joint training using both sentence-level and token-level features, and experimental results demonstrated that it achieved state-of-the-art (SOTA) performance on the corresponding dataset.

Beyond improving training effectiveness through deep learning, many researchers have further optimized NER models to accommodate the structural characteristics of Chinese electronic health records (EHRs). To address the challenges of word polysemy and incomplete word segmentation in Chinese EHR NER, Zhang et al. (2022) improved Chinese named entity recognition by combining the RoBERTa-WWM model with the BiLSTM-CRF model, and experimental results indicate that this model is better suited for Chinese EHR NER tasks. In order to cope with the problem of serious scarcity of annotated data in the medical field, Dao et al. (2024) proposed an entity-aware mask-based local fusion named entity recognition data enhancement EALMDA method, which increases sample diversity while preserving core semantics, thereby generating enhanced training samples, which significantly improves the effect compared with the mainstream baseline method of data enhancement. Shan et al. (2024) proposed a novel framework, NER-CMR, to address entity nesting and boundary recognition challenges in traditional NER, which consists of a character encoding module, word embedding module, graph construction module, fusion module, and CRF module, and was shown to outperform baseline models in recognition performance on the CCKS and DIABETES datasets.

On the other hand, to fully recognize and utilize knowledge in the medical domain—and to address the issue of polysemy in medical terminology—many researchers have proposed incorporating Knowledge Graphs (KGs) into medical named entity recognition. Hu et al. (2022) proposed a knowledge graph-inspired NER method, KGNER, which uses a masking and encoding strategy to incorporate common knowledge into the Transformer-based BERT model, enabling more effective use of external knowledge while preserving the semantic information of the original sentence; Jin et al. (2019) constructed the TCMKG-LSTM-CRF model to address the low recognition rate of rare words, which is commonly found in the field of traditional Chinese medicine, and used the knowledge graph information to enhance the model learning ability to identify rare words, in addition, the model introduces a knowledge-attention mechanism between neural network hidden vectors and candidate vectors from the knowledge graph, also considering the influence of the preceding word; Harnoune et al. (2021) proposed an end-to-end knowledge graph-based approach to extract and analyze information from biomedical clinical notes using the BERT model and CRF, effectively handling abstract biomedical concepts. Experiments showed that the method achieved a recognition accuracy of 90.7% on the Online PIPE dataset.

To fully leverage domain expertise in medicine, this paper proposes integrating the knowledge graph into the training process of the medical NER model by analysing the lexical relationship problem of the Chinese electronic medical record, strengthening the model’s understanding of the professional vocabulary in the medical field, by constructing a model that fuses the knowledge graph with the named entity recognition framework, and using the ClinicalBERT pre-training model (Huang et al., 2019) to obtain the text sequence of initial vector representations, where domain knowledge and entity relationships are encoded via knowledge graph embeddings. In addition, the proposed model utilizes a BiLSTM-CRF architecture to perform sequence modeling on the initial features, extracting contextual feature vectors to enhance representation learning, and finally decodes the optimal label sequences through CRF, which in turn improves the performance of the named entity recognition model.

Although previous studies have demonstrated the effectiveness of pre-trained models like BERT and BioBERT for clinical NER, these models are typically trained on general biomedical corpora and may lack adequate adaptation to the linguistic characteristics of real-world clinical narratives, such as abbreviations, incomplete sentences, and institution-specific terminology (Sun et al., 2021).

Moreover, most existing methods treat NER purely as a sequence labeling task, relying exclusively on token-level embeddings. They rarely leverage structured domain knowledge, which can be crucial for disambiguating similar entities and enhancing recognition performance in low-resource settings (Hu et al., 2022).

These limitations motivate our approach, which leverages ClinicalBERT for improved contextual representation of clinical texts and integrates a domain-specific medical knowledge graph to provide complementary semantic cues for the NER model (Zhou et al., 2021).

In recent years, large language models (LLMs), such as GPT-3, GPT-4, T5, and PaLM, have demonstrated remarkable performance in a wide range of natural language processing tasks, including few-shot and zero-shot named entity recognition (NER). By leveraging large-scale pre-training on diverse textual corpora, these models can generalize to downstream tasks with minimal supervision. Several studies have explored the application of LLMs to NER by reformulating the task as a generative problem or prompt-based classification. For instance, UIE (Unified Information Extraction) and PromptNER have shown that LLMs can perform information extraction without task-specific training, while maintaining strong flexibility and task adaptability.

In the biomedical domain, domain-adapted LLMs such as BioGPT, BioMedLM, and Med-PaLM have further extended the applicability of LLMs to clinical and biomedical NER. These models incorporate domain-specific corpora during pre-training and achieve promising results in zero-shot clinical concept extraction and question answering. However, the deployment of LLMs in medical NER still faces several challenges, including high computational cost, limited explainability, and difficulty in grounding predictions with structured external knowledge such as medical ontologies or knowledge graphs.

Therefore, this study deliberately adopts a lightweight and interpretable architecture based on ClinicalBERT and knowledge graph embeddings. This design enables better domain adaptation to Chinese EMRs, facilitates knowledge-guided entity disambiguation, and allows explicit integration of structured semantic constraints. While LLMs offer significant potential, future research may investigate hybrid paradigms that combine LLMs with structured domain knowledge to enhance performance and interpretability in clinical NER tasks.

## 3 Methodology

**Figure 1:**
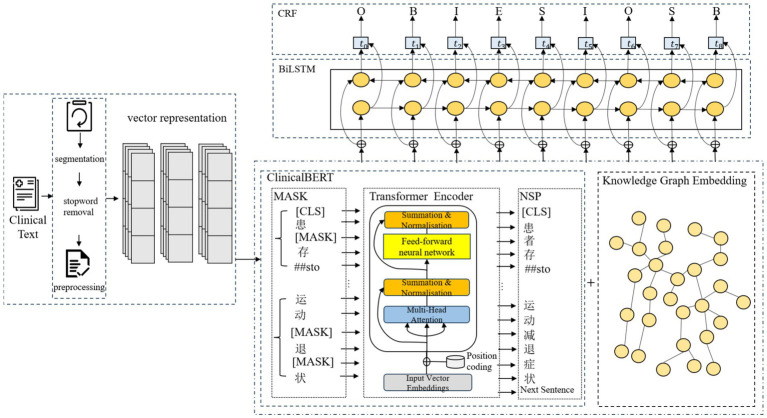
Named entity recognition model structure.

In this paper, based on the input representations generated by ClinicalBERT, we propose a Chinese electronic medical record named entity recognition model, CBT-KG, which integrates a knowledge graph and the ClinicalBERT pre-trained model, which mainly consists of a ClinicalBERT layer, a knowledge graph embedding layer, a BiLSTM layer, and a CRF layer. The overall structure is illustrated in Figure 1.

### 3.1 Text labelling and representation

We use a word segmentation tool to divide the text and apply the BIOES annotation scheme to mark the positions and entity categories within the sequence. The BIOES annotation scheme is more fine-grained than the traditional BIO format. In this scheme, the position labels are defined as follows B stands for the starting position of the entity (Begin), I stands for the inner part of the entity (Inside), O stands for the non-entity part (Outside), E stands for the ending position of the entity (End), and S stands for the entity consisting of a single word or character (Single). For entity category annotation, we adopt a two-layer label structure in the form of “position—entity_type,” tailored to electronic medical records BODY (Anatomical part), DRUG (Drugs), DISEASES (Diseases and diagnosis), EXAMINATIONS (Imaging examination), TEST (Laboratory test) and TREATMENT (Surgery), which are labeled as illustrated in Table 1.

**Table 1:** Example of labelling.

Character	Position	Entity type
无	O	O
胸	S	S-BODY
闷	O	O
胸	S	S-BODY
痛	O	O
胸	B	B-BODY
前	I	I-BODY
区	E	E-BODY
压	O	O
榨	O	O
感	O	O

To represent character-level features, we construct a character dictionary that indexes character attributes. Specifically, each character is labeled with its position, context category, and embedding features; then the corresponding feature embedding matrix is found according to the indexes corresponding to these labels, and these features are projected into a vector space. Finally, feature vectors from different dimensions are concatenated to form a comprehensive representation for each character. Let the input sequence be $S=\{x_{1},x_{2},\ldots ,x_{n}\}$ , in which the label corresponding to character $c_{i}$ is vector-mapped to obtain the positional feature $p_{i}\in R^{d}$ , the contextual category feature $t_{i}\in R^{d}$ , and the embedding feature $a_{i}\in R^{d}$ . The multidimensional feature vector corresponding to each character $c_{i}$ can be obtained by stitching the above three feature vectors element by element, and the calculation is shown in Equation 1:


(1) $$x_{i}=\text{Concat}(p_{i},t_{i},a_{i})$$

### 3.2 ClinicalBERT module

BERT is a bidirectional Transformer model that excels in natural language processing tasks through large-scale unsupervised pre-training and supervised fine-tuning. The Transformer consists of multi-layered encoders and decoders. Each encoder includes a multi-head attention mechanism, a feed-forward neural network, residual connections, and layer normalization. The pre-training objective function of the ClinicalBERT model is defined by two unsupervised tasks, masked language modelling and next sentence prediction.

**Figure 2:**
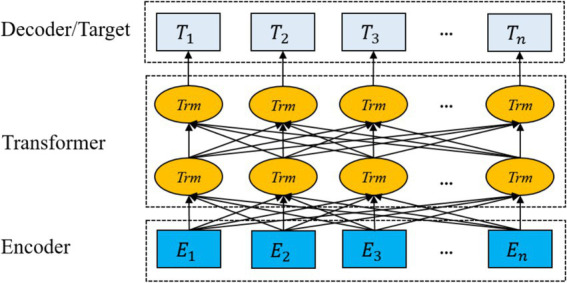
ClinicalBERT structure.

However, the pre-training data of standard BERT models (e.g., Wikipedia and BookCorpus) do not cover the terminology, syntax, and context specific to the medical domain. To address this issue, ClinicalBERT conducts domain-specific pre-training based on BERT using medical texts such as electronic health records, clinical notes, and medical literature, enabling it to better understand linguistic expressions in the medical domain, such as diagnostic terminology, treatment plans, and drug names; In addition, ClinicalBERT is well-suited for handling clinical texts that contain specialized terminology and complex medical syntax. It inherits the advantages of dynamic contextual representations from BERT, enabling it to better handle word sense ambiguity compared to traditional static embedding methods. Traditional static embedding methods only focus on the word itself and the local features of its location, making it difficult to handle word polysemy. In contrast, ClinicalBERT leverages bidirectional semantics, focuses on different contextual information through multi-head self-attention mechanisms across multiple encoder layers and calculates dependency relationships between words within a sentence, thereby assigning different weights to each word. As a result, the same word receives different vector representations in varying contexts, which effectively solves the problem of word sense ambiguity. The architecture of the ClinicalBERT model is illustrated in Figure 2.

ClinicalBERT primarily relies on multi-head attention mechanisms to compute input vectors, as shown in Equation 2:


(2) $$\text{Attention}\;(Q,K,V)=\text{soft}\max(\frac{QK^{T}}{\sqrt{d}}V)$$

where Q , K , and V refer to the query, key, and value vectors, respectively, and d refers to the dimensions of the query, key, and value vectors, Q is used to perform dot product operation on each key vector, and the result is scaled and normalised by softmax function to obtain the attention weight matrix. Specifically, the multi-head attention mechanism uses multiple parallel attention heads to capture different feature representations at the same time, and each head computes self-attention in different subspaces to make the model pay attention to different input information from multiple perspectives, and finally the heads are spliced together and linearly transformed to obtain the output of the feature matrix that integrates the attention information, which is computed as shown in Equations 3–5.


(3) $${\text{head}}_{h}=\text{Attention}(Q^{\left(h\right)},K^{\left(h\right)},V^{\left(h\right)})$$

(4) $$\text{MultiHeadOutput}=\text{Concat}(\mathrm{hea}d_{1},\mathrm{hea}d_{2},\ldots ,{\text{head}}_{h})$$

(5) $$\text{Output}=\text{Concat}(\mathrm{hea}d_{1},\mathrm{hea}d_{2},\ldots ,{\text{head}}_{h})W_{o}$$

where $W_{o}$ is the output weight matrix.

**Figure 3:**
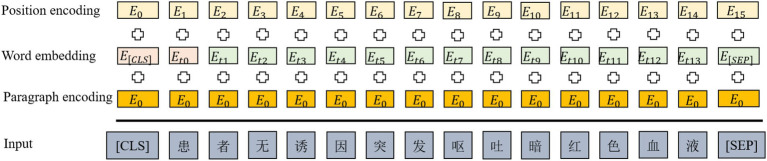
ClinicalBERT input style.

In the input phase of the ClinicalBERT model, each input sentence is represented as the summation of three embeddings: token embeddings, positional embeddings, and segment embeddings. A special token [CLS] is added at the beginning of the sentence to represent the overall sequence, while [SEP] is inserted at the end to indicate sentence boundaries or separate multiple segments (see Figure 3).

**Figure 4:**
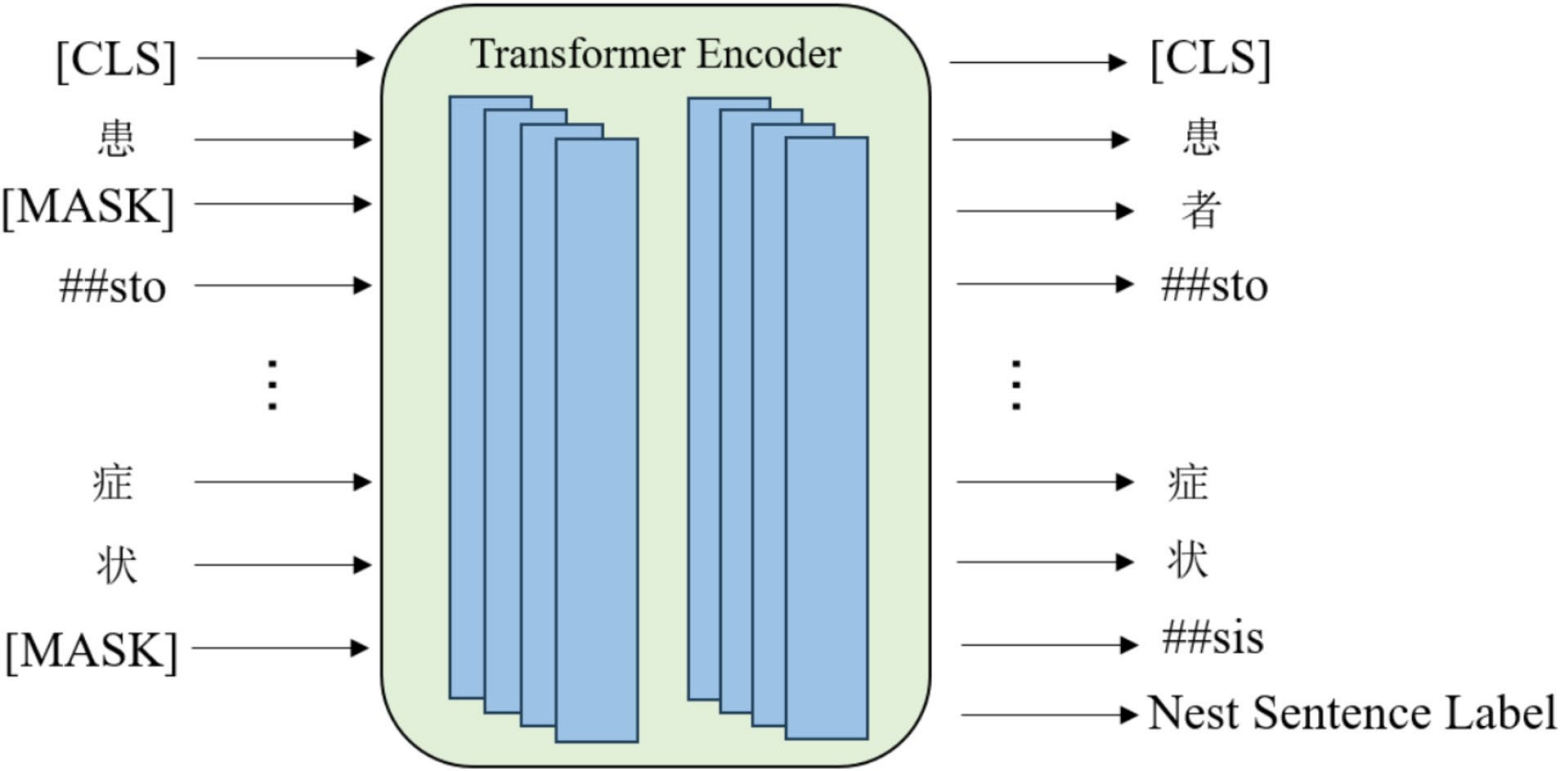
ClinicalBERT pre-training process.

The ClinicalBERT model adopts two unsupervised tasks, namely Masked Language Modelling and Next Sentence Prediction, to pre-train the model, and in addition, by incorporating the knowledge of medical text in the pre-training process, the performance of the model in the recognition of named entities in medical-related domains is greatly improved. Domain named entity recognition by incorporating medical text-related knowledge in the pre-training process, and the pre-training process is shown in Figure 4.

Figure 4 illustrates the pre-training process of ClinicalBERT, which is based on the standard BERT architecture but trained specifically on clinical narratives. The input text is tokenized and embedded using WordPiece, and special tokens such as [CLS], [SEP], and [MASK] are inserted. Two training objectives are employed: masked language modeling (MLM), where certain tokens (e.g., “acute,” “##sto”) are randomly masked and the model learns to predict them based on context; and next sentence prediction (NSP), where the model predicts whether one sentence logically follows another.

Unlike general-purpose BERT models, ClinicalBERT is pre-trained on de-identified clinical notes from MIMIC-III, enabling it to better understand the unique characteristics of medical language, such as frequent abbreviations, fragmented grammar, and domain-specific terminology. This domain adaptation enhances the model’s effectiveness in downstream clinical NLP tasks, including named entity recognition and clinical relation extraction.

### 3.3 Knowledge graph embedding module

#### 3.3.1 Building the knowledge graph

A Knowledge Graph is a graph-based structure consisting of nodes (entities) and edges (relationships). As a structured semantic knowledge base, it serves as a critical foundation for many artificial intelligence applications (Zhao et al., 2024). In the field of medicine, it usually includes entities such as diseases, drugs, body parts, symptoms, and treatments as well as relationships such as “treat” and “cause.”

To enhance the performance of the NER model, we constructed a domain-specific medical knowledge graph using a semi-automatic pipeline. The sources of knowledge include publicly available and widely used datasets such as MIMIC-III discharge summaries, DrugBank, and UMLS. These sources ensure that the extracted entities and relations are of high relevance and consistency.

**Figure 5:**
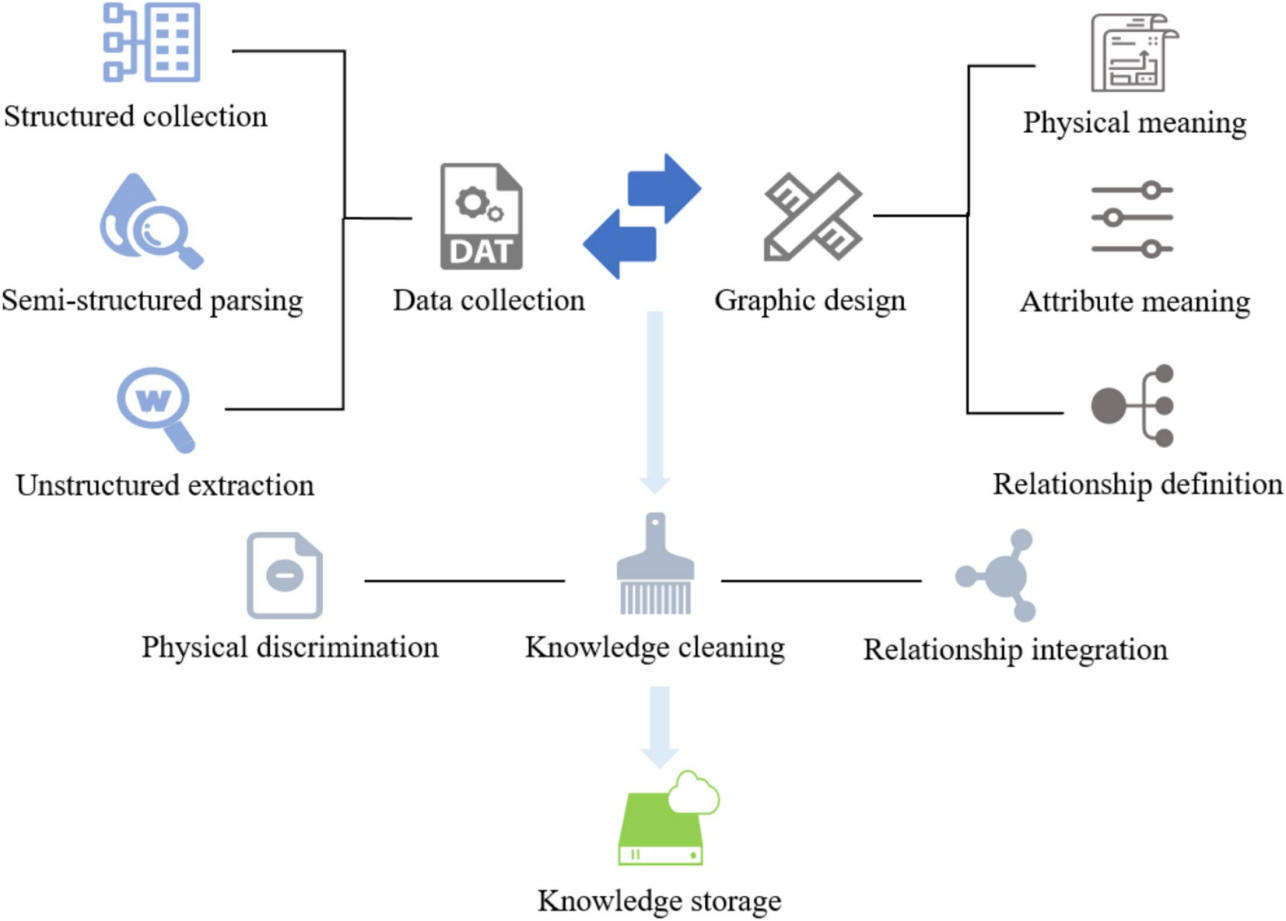
The process of constructing a knowledge graph.

The knowledge graph contains seven types of entities—Check, Department, Disease, Drug, Food, Producer, and Symptom—as well as several semantic relations such as “belongs_to,” “common_drug,” “drugs_of,” “need_check,” “recommend_drug,” “has_symptom,” and “accompany_with.” For instance, the triple (“Hypertension,” “recommend_drug,” “Captopril”) represents a common therapeutic relation. In total, 28,765 entities and 63,182 relations were identified, resulting in approximately 95,000 triples. The detailed construction process is illustrated in Figure 5.

To enhance the quality of the constructed medical knowledge graph, we employed a set of rule-based and resource-guided strategies to reduce ambiguity and noise. Synonym normalization was performed using UMLS, SNOMED CT, and DrugBank, supplemented by a manually curated dictionary, to unify variants such as “MI” and “myocardial infarction.” Abbreviations were expanded using context-aware rules based on co-occurring terms and section information within clinical notes. Low-frequency entities and relations were pruned based on an empirically determined threshold to eliminate unreliable triples. Finally, each triple was cross-validated against multiple trusted sources, and only those confirmed by at least two databases were retained, ensuring the semantic accuracy and clinical reliability of the graph.

**Figure 6:**
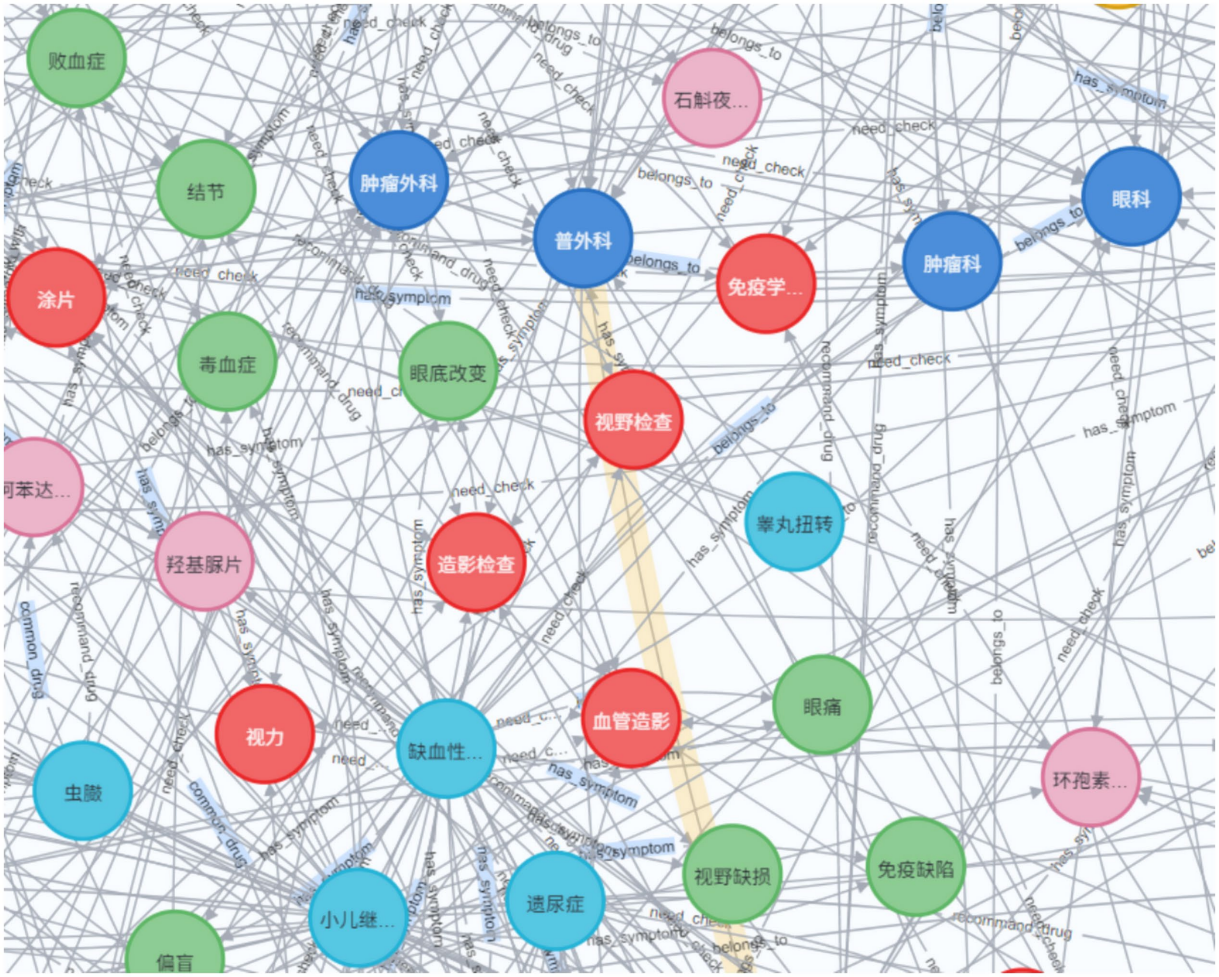
Example of a knowledge graph (partial).

The knowledge graph constructed in this paper contains seven types of entity types and entity relationship types, whose names and Chinese meanings are shown in Table 2. The neo4j graph database is used as a visualization tool, and its local effect is shown in Figure 6.

**Table 2:** Entities and entity relationship types.

Entity type	Chinese meaning	Type of entity relationship	Chinese meaning
Check	诊断检查项目	belongs_to	属于
Department	医疗科室	common_drug	常用药品
Disease	疾病	drugs_of	在售药品
Drug	药品	need_check	疾病所需检查
Food	食物	recommend_drug	推荐药品
Producer	在售药品	has_symptom	疾病症状
Symptom	疾病症状	accompany_with	并发疾病

#### 3.3.2 Knowledge graph embedding

Knowledge graph embedding (KGE) is the process of constructing entities and relationships within a knowledge graph in a low-dimensional vector space, which facilitates downstream tasks to capture complex semantic relationships in the knowledge graph more efficiently and accurately (Yan et al., 2022), and is fundamental research in important areas such as semantic retrieval, knowledge query and recommendation. Integrating knowledge graph embeddings into medical NER tasks can enhance model accuracy, extend recognition coverage, improve performance in low-resource scenarios, and provide semantic understanding and reasoning capabilities to better support clinical decision-making and medical research.

Knowledge graph embedding methods usually consist of simple embedding methods based on embedding lookup and classical KGE methods, the former relying on entity ID mapping to pre-trained embedding vectors to achieve knowledge graph embedding and integrating it into downstream tasks by splicing with text embedding. The method is simple to implement and suitable for rapid integration into existing models, in addition to being computationally efficient as it only requires lookup operations to obtain the embedding vectors of entities. However, the method lacks relational modelling and does not explicitly model the relationships between entities, which may result in the model failing to effectively capture the complex semantic associations between entities. On the other hand, the method may not be fully adapted to the needs of downstream tasks since the embeddings of the entities are pre-trained and remain unchanged throughout the training process without fine-tuning.

Bordes et al. (2013) proposed TransE (Translation Embedding) knowledge graph embedding method for embedding entities and relations from multi-relational data into low-dimensional vector spaces. Therefore, the introduction of TransE method to embed knowledge graphs can effectively utilize the structural information of knowledge graphs to enhance the performance of models. TransE assumes that there exists a triple (h,r,t) in the knowledge graph, and the core idea is to map head entities, relations and tail entities into a continuous vector space by translation, such that the triple satisfies the following equation, as shown in Equation 6:


(6) h+r≈t

where h is the vector representation of the head entity, r is the vector representation of the relation, and t is the vector representation of the tail entity. Specifically, TransE models the relationships in the knowledge graph as displacements between the head and tail entities by means of a translation operation, and for a correct ternary (h,r,t) , the TransE model is to minimise the distance function f(h,r,t) , as shown in Equation 7:


(7) $$f(h,r,t)={\left\|h+r-t\right\|}_{p}$$

where ${\left\|•\right\|}_{p}$ is the L1-paradigm (absolute sum) or L2-paradigm (Euclidean distance) of the vector, and the smaller the value of f(h,r,t) , the more valid the triad (h,r,t) is.

The core goal of TransE is to preserve the structural information in the knowledge graph by optimising the loss function so that the embedding vectors of the correct triples (*h*, *r*, *t*) are closer together and the wrong triples, the negative samples, are farther away. TransE is optimised using a margin-based ranking loss function, which is formulated as Equation 8:


(8) $$L=\sum_{(h,r,t)\in \tau }\sum_{(h\prime ,r\prime ,t\prime )\in \tau \prime }{\left[\gamma +f(h,r,t)-f(h\prime ,r\prime ,t\prime )\right]}_{+}$$

where τ represents the set of positive samples; τ′ represents the set of negative samples, which is usually generated by randomly replacing h or t ; γ is the Margin hyperparameter, which controls the spacing between the positive and negative samples; and ${\left[x\right]}_{+}=\max(0,x)$ is a ReLU operation that guarantees a non-negative loss.

### 3.4 Integration of ClinicalBERT and knowledge graph embeddings

To enable joint modeling of contextual semantics and structured domain knowledge, this study adopts an early fusion strategy that integrates the hidden representations produced by ClinicalBERT with entity embeddings derived from a medical knowledge graph. After tokenizing a Chinese electronic medical record at the character level, the input sequence is fed into ClinicalBERT to produce contextual embeddings $\left\{h_{1}^{\text{BERT}},h_{2}^{\text{BERT}},\ldots ,h_{n}^{\text{BERT}}\right\}$ , where $h_{i}^{\text{BERT}}\in {\mathbb{R}}^{d}$ denotes the output for the *i*-th character.

Concurrently, an entity linking step is performed to associate each character (or character span) with its corresponding medical entity in the knowledge graph. For each matched entity, a semantic embedding $e_{i}^{\mathrm{KG}}\in {\mathbb{R}}^{d\prime }$ is retrieved, which is pre-trained using the TransE algorithm. To ensure dimensional consistency with the ClinicalBERT output space, each knowledge embedding is passed through a learnable linear projection, as shown in Equation 9:


(9) $${\tilde{e}}_{i}^{\mathrm{KG}}=W_{e}e_{i}^{\mathrm{KG}}+b_{e},{\tilde{e}}_{i}^{\mathrm{KG}}\in {\mathbb{R}}^{d}$$

The projected knowledge vector ${\tilde{e}}_{i}^{\mathrm{KG}}$ is then concatenated with the contextual embedding $h_{i}^{\text{BERT}}$ to form a joint representation, as shown in Equation 10:


(10) $$h_{i}^{\mathrm{jo}\mathrm{int}}=[h_{i}^{\text{BERT}};{\tilde{e}}_{i}^{\mathrm{KG}}]\in {\mathbb{R}}^{2d}$$

For characters that are not linked to any entity in the knowledge graph, a zero vector or learned placeholder embedding is used to substitute $e_{i}^{\mathrm{KG}}$ , ensuring that all characters contribute to the same fused sequence. The final joint representation sequence ${\left\{h_{i}^{\mathrm{jo}\mathrm{int}}\right\}}_{i=1}^{n}$ is passed into a BiLSTM layer to model bidirectional sequential dependencies, and the optional label sequence is decoded using a CRF layer.

This integration mechanism allows the model to simultaneously leverage the local contextual understanding from ClinicalBERT and the structured domain semantics from the knowledge graph. For example, in the sentence “患者出现糖尿病视网膜病变” (“the patient exhibited diabetic retinopathy”), ClinicalBERT captures the immediate context surrounding the term “糖尿病,” while the knowledge embedding conveys that this entity is a chronic metabolic disease with established links to ocular complications such as “视网膜病变.” This complementary fusion strengthens the model’s ability to disambiguate semantically similar terms, identify rare entities, and improve overall recognition performance, particularly in complex and noisy clinical narratives.

### 3.5 BiLSTM-CRF module

To effectively capture contextual dependencies in clinical text and ensure global consistency of predicted labels, this study integrates Bidirectional Long Short-Term Memory (BiLSTM) networks with a Conditional Random Field (CRF) layer. The BiLSTM captures both forward and backward contextual information, enhancing the semantic understanding of medical terminology, while the CRF layer optimizes the label sequence by modeling dependencies between adjacent tags.

### 3.6 BiLSTM encoding

While standard LSTM networks alleviate vanishing and exploding gradient issues through gating mechanisms, they process sequences in a single direction. In contrast, BiLSTM employs two LSTM networks to process the input sequence in both forward and backward directions, allowing it to simultaneously capture past and future information. This is particularly beneficial for modeling the unstructured and complex nature of electronic medical records (EMRs). After inputting the initial vector representation E into the BiLSTM layer, the formula of the forward LSTM module is shown as Equations 11–13:


(11) $$\left[\begin{array}{l} i_{t}\\ o_{t}\\ f_{t}\\ {\tilde{c}}_{t} \end{array}]=[\begin{array}{l} \sigma \\ \sigma \\ \sigma \\ \tanh \end{array}](W[\begin{array}{l} E\\ h_{t-1} \end{array}]+b\right)$$

(12) $$c_{t}=f_{t}⊙c_{t-1}+i_{t}⊙{\tilde{c}}_{t}$$

(13) $$h_{t}=o_{t}⊙\tanh(c_{t})$$

Finally, the output vectors of the forward and backward LSTMs are spliced together as the output of the BiLSTM neural network, as shown in Equation 14:


(14) $$h_{t}=[\vec{h_{t}};\overset{\leftarrow }{h_{t}}]$$

#### 3.6.1 CRF decoding

Although Transformer-based models such as ClinicalBERT can generate rich semantic representations, they typically predict labels independently and fail to model dependencies among output tags, which may result in invalid label sequences. To address this issue, we apply a CRF layer on top of the BiLSTM outputs to capture the correlations between adjacent labels and ensure valid transitions. Given an input sequence X and a label sequence Y , the conditional probability defined by CRF is shown as Equation 15:


(15) $$P(Y|X)=\frac{1}{Z(X)}\exp(\sum_{i-1}^{n}\sum_{j-1}^{k}\omega_{j}•f_{j}(x_{i},y_{i-1},y_{i}))$$

where P(Y∣X) denotes the probability of occurrence of the output sequence Y given the input sequence X ; Z(X) is the normalization factor over all possible label sequences, the calculation is shown as Equation 16:


(16) $$Z(X)=\sum_{Y}\exp(\sum_{i=1}^{n}\sum_{j-1}^{k}\omega_{j}•f_{j}(x_{i},y_{i-1},y_{i}))$$

where Y denotes all possible combinations of label sequences; $\omega_{j}$ is a model parameter used to assign the corresponding weight to the feature function $f_{j}(x_{i},y_{i-1},y_{i})$ and k is the total number of feature functions.

The CRF parameters are learned by maximizing the log-likelihood of the correct label sequences. During inference, the optimal label path is obtained using the Viterbi algorithm.

The BiLSTM-CRF architecture effectively combines contextual semantic encoding with global sequence optimization, yielding more accurate and consistent recognition of medical entities.

## 4 Experiments

### 4.1 Datasets

The experiments in this paper use the electronic medical record datasets CCKS2019 and CCKS2020, which were released by the China conference on knowledge graph and semantic computing (CCKS) in 2019 and 2020. Each medical record data contains raw text and predefined entity labels, and the datasets are labelled with a total of six entity types, namely Disease and Diagnosis (Dis), Drug, Surgery (Operation), Imaging Exam (ImgExam), Anatomical Sites (Anatomy), and Laboratory Exam (LabExam). The CCKS2019 dataset contains 1,379 pieces of data, of which 1,000 data for training and 379 data for testing; CCKS2020 dataset contains 1,500 data, of which 1,200 data for training and 300 data for testing, and the statistics of the number of each entity type in the training and testing sets of the two datasets are shown in Table 3.

**Table 3:** Types and number of data entities.

Entity type	CCKS2019		CCKS2020	
	Training set	Test set	Training set	Test set
Dis	3,645	1,808	6,211	1,361
Operation	908	162	1,327	221
Drug	1,593	485	2,841	942
Anatomy	7,158	3,094	12,660	2,661
ImgExam	888	348	1,490	270
LabExam	991	590	1,885	251

### 4.2 Evaluation metrics

The experiments used Precision, Recall and *F*1 values to evaluate the recognition effectiveness of the model, which are calculated as Equations 17–19:


(17) $$P=\frac{\mathrm{TP}}{\mathrm{TP}+\mathrm{FP}}\times 100\%$$

(18) $$R=\frac{\mathrm{TP}}{\mathrm{TP}+\mathrm{FN}}\times 100\%$$

(19) $$F1=\frac{2\times P\times R}{P+R}\times 100\%$$

where: TP denotes the number of correctly identified positive samples; FP denotes the number of negative samples incorrectly identified as positive samples; and FN denotes the number of positive samples incorrectly identified as negative samples. P denotes the proportion of correctly predicted results to the total number of predicted results; R denotes the proportion of correctly predicted results in all the data; and F1 is the reconciled mean of P and R .

### 4.3 Experimental settings

Before the experiments, the maximum length of the sentences was set to the maximum length of 512 supported by ClinicalBERT using the BIOES annotation method, and the sentences exceeding this length were added to the dataset after being cut. The experiments were conducted using python3.8.19, pytorch2.3.1 framework, and cuda11.8 to train the model, while the model parameters were fine-tuned during the experiments, and the model with the best performance on the validation set was finally chosen to evaluate the test set.

The detailed parameters of the models in this paper are shown in Table 4.

**Table 4:** Parameter list.

Hyperparameter	Value
Batch Size	16
Learning rate	$1.0\times 10^{-4}$
Dropout	0.5
Optimiser	Adam
Epoch	100
Hidden layers	256
BiLSTM layers	2

### 4.4 Discussion

#### 4.4.1 Model performance analysis

In order to validate the effectiveness of the model proposed in this paper, the experiments are selected to compare and analyse some of the baseline models commonly used in the field of named entity recognition, the experiments are firstly conducted on the dataset CCKS2019, and the results obtained from the experiments are shown in Table 5.

**Table 5:** CCKS2019 experimental data.

Models	*P*/%	*R*/%	*F*1/%
BiLSTM-CRF	80.45	80.37	81.92
Lattice-LSTM (Zhang and Yang, 2018)	79.52	81.59	82.12
FLAT (Li et al., 2020)	81.56	83.34	83.55
BERT-BiLSTM-CRF (Dai et al., 2019)	81.35	84.23	84.75
ME-NER (Xu et al., 2019)	83.49	82.57	83.24
MacBERT (Cui et al., 2020)	87.28	84.52	86.45
PL-Marker (Falk and Lapesa, 2022)	88.59	84.59	87.27
CBT-KG	**90.47**	**88.37**	**89.44**

The bold values indicate the best performance for each metric.

To further prove the effectiveness of the proposed model in this paper, the CCKS2020 dataset is also selected for experiments, and its experimental results are shown in Table 6.

**Table 6:** CCKS2020 experimental data.

Models	*P*/%	*R*/%	*F*1/%
BLSTM-CRF	81.88	84.23	83.12
Lattice-LSTM (Zhang and Yang, 2018)	84.25	85.96	85.08
FLAT (Li et al., 2020)	85.67	87.79	86.72
BERT-BiLSTM-CRF (Dai et al., 2019)	86.25	88.50	87.54
ME-NER (Xu et al., 2019)	87.59	89.14	88.32
MacBERT (Cui et al., 2020)	88.22	87.16	85.49
PL-Marker (Falk and Lapesa, 2022)	87.29	88.53	87.95
CBT-KG	**90.69**	**89.31**	**89.29**

The bold values indicate the best performance for each metric.

From the experimental data in Tables 5, 6, it can be seen that the *F*1 value of the model proposed in this paper reaches 90.44% on the CCKS2019 dataset, which improves the overall effect from 4.69 to 7.52% compared with the other benchmark models; and the *F*1 value on the CCKS2020 dataset reaches 89.29%, which improves the overall effect from 0.97 to 6.17%, which can prove the effectiveness of the proposed model in the Chinese EHR named entity recognition task. The experimental results are analyzed as follows:

Among the baseline models, BiLSTM-CRF effectively integrates contextual information and global label dependencies, but its ability to represent complex semantics is limited. When incorporating BERT as a pre-trained encoder, the BERT-BiLSTM-CRF model shows substantial gains in *F*1 scores, benefiting from BERT’s bidirectional contextual encoding and deep semantic representation capabilities. These enhancements help the model better capture long-distance dependencies and ambiguous expressions in clinical narratives.

The Lattice-LSTM model further improves performance by introducing a lattice structure to integrate character-level and word-level features. This structure enables multi-granular representation and enhances disambiguation in Chinese sequences, especially for ambiguous or nested entities. However, its performance still lags behind BERT-based models, likely due to its limited semantic modeling capacity compared to large-scale pre-trained language models.

Building upon Lattice-LSTM, FLAT replaces the lattice structure with span-based encoding and introduces relative position representations via self-attention mechanisms. These enhancements yield further performance improvements by enabling more effective modeling of long text spans and flexible contextual relationships.

Our proposed model differs fundamentally from these approaches by incorporating both domain-specific contextual modeling and structured external knowledge. ClinicalBERT, pre-trained on clinical corpora, offers improved understanding of medical terminology and fragmented clinical syntax, making it more suitable for EMR texts than general-purpose BERT variants. In parallel, we embed structured semantics from a curated medical knowledge graph using TransE-based embeddings. This integration enriches the model with domain knowledge, improving its ability to disambiguate similar entities and capture inter-entity relationships, which general-purpose pre-trained models cannot readily provide.

Recent models such as MacBERT and PL-Marker have shown promise in general-domain NER tasks, with improved masking strategies and prompt-based formulations. However, these methods are trained primarily on general-purpose corpora and lack adaptation to clinical language, where challenges like medical abbreviations, domain-specific vocabulary, and irregular syntax are prevalent. In contrast, our model is explicitly designed for clinical text by combining ClinicalBERT with a structured medical knowledge graph, demonstrating clear superiority on both datasets.

In summary, while traditional models emphasize internal linguistic patterns or span-based encoding strategies, our approach bridges contextual learning and external domain knowledge. This synergy leads to the most consistent and significant improvements across all baselines, underscoring the effectiveness of combining ClinicalBERT and knowledge graph embeddings in the medical NER task.

Although the current experimental setup compares CBT KG with widely adopted and strong baseline models such as BiLSTM CRF, BERT BiLSTM CRF, MacBERT, and PL Marker, we acknowledge that a direct comparison with more recent knowledge enhanced named entity recognition models such as KALA, LUKE, KnowPrompt was not included. This is primarily because most of these advanced models lack available Chinese medical domain adaptations or open source implementations that support integration with structured knowledge resources like the medical knowledge graph developed in this study. Furthermore, many of these models are designed and evaluated on general domain or English language datasets, such as CoNLL 2003 and TACRED, which makes fair and controlled comparisons within the context of Chinese clinical named entity recognition nontrivial.

Nevertheless, we acknowledge the importance of placing CBT KG within the broader context of recent knowledge enhanced NER research. In future studies, we intend to explore the integration of newly developed large scale knowledge based models and prompt driven learning approaches into our framework. We also plan to assess their effectiveness on Chinese electronic medical record datasets. Such efforts will provide further evidence of the flexibility and potential of CBT KG when applied to more complex and varied clinical scenarios.

#### 4.4.2 Comparison of knowledge graph embedding methods

To further evaluate the impact of different knowledge graph embedding strategies on the performance of medical NER, we compared three representative translation-based embedding models: TransE, TransH, and TransR. These methods were chosen for their wide adoption and conceptual diversity, allowing us to assess how varying levels of modeling complexity affect the downstream entity recognition task. The results of the experiments are shown in Table 7.

**Table 7:** Comparison of knowledge graph embedding methods (on CCKS2019).

KGE method	*P*/%	*R*/%	*F*1/%
TransE	**90.47**	**88.37**	**89.44**
TransH	89.82	87.94	88.86
TransR	90.25	88.12	89.17
Without KG	89.60	84.64	85.49

The bold values indicate the best performance for each metric.

TransE, the simplest among the three, treats relations as translation operations in the embedding space. Given a triple (head, relation, tail), it learns embeddings such that the vector of the tail entity is close to the head entity plus the relation vector. This method is computationally efficient and well-suited for large-scale or moderately structured graphs. In our experiments, the TransE-based model achieved the highest *F*1 score (89.44%), demonstrating that even a basic geometric representation of relations is sufficient to enhance entity disambiguation when combined with ClinicalBERT.

TransH extends TransE by allowing entities to have different representations when involved in different relations. It introduces a relation-specific hyperplane to better handle one-to-many, many-to-one, and many-to-many relationships. However, in our experiments, the TransH-based model achieved a slightly lower *F*1 score (88.86%). Although theoretically more expressive, TransH may overfit small or moderately sized graphs due to its increased parameterization, which is consistent with prior findings that complex embeddings can degrade performance when relational density is low.

TransR further increases modeling capacity by projecting entities and relations into distinct vector spaces. Each relation has an associated projection matrix, enabling it to model heterogeneous interactions more flexibly. Despite this, the TransR variant yielded only marginal improvement over TransH (*F*1 = 89.17%) and did not surpass TransE. The additional complexity introduced by relation-specific matrices led to slower convergence and greater sensitivity to hyperparameter tuning. This suggests that the expressiveness of TransR may not yield proportional performance gains for clinical NER tasks grounded in relatively simple ontological structures.

In summary, while all three KGE methods improved performance over the non-KG baseline (*F*1 = 85.49%), TransE demonstrated the best trade-off between representational adequacy, computational efficiency, and model generalizability in our setting. These results reinforce the observation that lightweight and interpretable embedding strategies are often more suitable for integration into language models targeting clinical applications, particularly when the knowledge graph is carefully curated and domain-specific.

#### 4.4.3 Ablation experiments

In order to verify the impact of each module of the model on the overall performance of the model, this paper designs ablation experiments, mainly exploring the impact of the embedding of the knowledge graph on the model to perform the task of recognising named entities in Chinese EHRs, and the results of the experiments are shown in Table 8.

**Table 8:** Results of ablation experiments.

Model	CCKS2019			CCKS2020		
	*P*/%	*R*/%	*F*1/%	*P*/%	*R*/%	*F*1/%
CBT-KG	**90.47**	**88.37**	**89.44**	**90.69**	**89.31**	**89.29**
CBT (w/o KG)	89.60	84.64	85.49	88.45	84.26	84.79

The bold values indicate the best performance for each metric.

The following analysis can be derived from the data in Table 8:

The CBT-KG model incorporates medical knowledge graph embedding compared to the CBT model, and the knowledge graph embedded in the named entity recognition model can enhance the model’s ability to understand medical terminology and complex semantic relationships, which in turn improves the model’s recognition performance in the task of recognizing named entities in Chinese electronic medical records. From the experimental results, it can be seen that the model with the addition of knowledge graph embedding improves the accuracy, recall and *F*1 value by 0.87, 3.73 and 3.95% on the CCKS2019 dataset, and by 2.24, 5.05 and 4.5% on the CCKS2020 dataset compared with the model without knowledge graph embedding, respectively. This demonstrates the effectiveness of knowledge graph embedding for improving the model named entity recognition performance.

To investigate the individual contributions of the multi-source character-level features described in Section 3.1, namely, BIOES positional tags, contextual category features, and semantic embedding vectors, an ablation study was conducted by selectively removing each component from the input representation while keeping the other two fixed. The results, summarized in Table 9, reveal that all three feature types contribute significantly to the model’s overall performance, with varying degrees of impact.

**Table 9:** Contribution analysis of character-level features (on CCKS2019).

Model variant	*P*/%	*R*/%	*F*1/%
Full multi-source features	**90.47**	**88.37**	**89.44**
w/o BIOES positional features	89.32	86.95	88.12
w/o contextual category features	88.90	86.13	87.50
w/o embedding semantic vectors	87.45	85.09	86.26

The bold values indicate the best performance for each metric.

The ablation results indicate that all three character-level features contribute substantially to model performance, with semantic embedding vectors having the greatest impact, a 3.18% drop in *F*1 when removed. These vectors provide domain-level semantic cues from the knowledge graph, aiding in the disambiguation of rare or ambiguous entities. Removing BIOES positional tags leads to a 1.32% *F*1 decrease, as these tags guide entity boundary recognition in Chinese texts lacking explicit word delimiters. The absence of contextual category features results in a 1.94% performance drop, reflecting their role in refining entity type classification based on linguistic patterns. Overall, the features offer complementary benefits in semantic disambiguation, boundary detection, and type prediction, jointly enhancing NER robustness in clinical narratives.

#### 4.4.4 Error analysis

To further understand the limitations of the proposed model, we performed a qualitative analysis of its prediction errors on the CCKS2020 test set. One recurring issue lies in the inaccurate identification of entity boundaries, especially for multi-character medical terms. For instance, in the phrase “右侧肾上腺腺瘤可能性大” (“high likelihood of right adrenal adenoma”), the model sometimes extracts only “腺瘤” (“adenoma”), omitting the preceding anatomical modifier “右侧肾上腺” (“right adrenal”). This results in semantically incomplete entities and underscores the challenges posed by the lack of explicit word delimiters in Chinese clinical text, as well as the complexity of nested or compound expressions.

Another common source of error involves confusion between semantically similar entity types. In some cases, entities such as “心包积液” (“pericardial effusion”) are misclassified as symptoms rather than diagnoses. These errors suggest that, although the model incorporates contextual signals and external knowledge, it still struggles with fine-grained semantic distinctions across entity categories. In addition, recognition performance tends to degrade for rare or unseen medical terms that are not present in the knowledge graph. For example, “库欣综合征” (“Cushing’s syndrome”) was occasionally missed or incorrectly labeled, likely due to the absence of corresponding entity embeddings. This reveals the model’s reliance on the coverage and quality of the external knowledge base.

These findings reflect ongoing challenges in accurate boundary detection, semantic disambiguation, and robust handling of low-frequency medical terms. Future efforts may focus on integrating span-based decoding strategies, confidence-aware classification, or dynamically updating knowledge representations to further enhance the model’s precision and generalizability in clinical named entity recognition tasks.

#### 4.4.5 Computational considerations

To assess the practical feasibility of the proposed CBT KG model in clinical scenarios, we report its computational performance during training and inference. All experiments were conducted on a workstation equipped with an NVIDIA RTX 2080 GPU with 8 gigabytes of memory. The training process required approximately 9 h to converge on the CCKS2019 dataset and 7.5 h on the CCKS2020 dataset, with a batch size of 16. During inference, the model achieved an average processing speed of approximately 30 records per second in batch mode. Although the integration of ClinicalBERT, knowledge graph embeddings, BiLSTM, and CRF introduces additional computational overhead compared to simpler baseline models, the observed inference speed is acceptable for offline or periodic electronic medical record processing. For real-time deployment or resource-constrained clinical environments, further optimization techniques such as knowledge distillation, model pruning, or architecture simplification may be explored. These results suggest that the CBT KG model is compatible with typical GPU settings in hospital information systems and can serve as a foundation for practical clinical NLP applications.

#### 4.4.6 Impact of knowledge graph quality on NER performance

While the integration of a structured medical knowledge graph significantly enhances entity recognition, it also introduces potential vulnerabilities related to the quality of the graph itself. The synonym normalization and abbreviation expansion steps used in the construction process, although effective in increasing coverage, may inadvertently introduce noisy or incorrect triples due to rule-based heuristics or incomplete dictionaries. These inaccuracies can propagate through the model by introducing misleading semantic signals, ultimately impacting the reliability of entity classification and boundary detection.

In the context of clinical applications, where high accuracy is essential, this risk of error propagation cannot be overlooked. To address this concern, future versions of the model could incorporate strategies for noise-aware knowledge integration. For instance, assigning confidence scores to triples based on their source reliability or semantic coherence can help filter out less trustworthy information. Additionally, incorporating consistency checks or embedding-space denoising techniques may further reduce the impact of spurious knowledge. Dynamic fine-tuning of knowledge embeddings alongside the NER objective is another promising direction, allowing the model to adjust the influence of external knowledge in task-specific contexts. These strategies can enhance model robustness, ensuring safer deployment in real-world clinical scenarios.

#### 4.4.7 Multilingual adaptability and generalization potential

This study is designed specifically for Chinese electronic medical records, which pose distinctive challenges due to the absence of clear word boundaries and the complexity of clinical language. These factors influenced our decision to adopt character-level modeling and integrate structured domain knowledge into the model. However, we recognize that this focus may limit direct application to other languages, and a broader discussion on generalization is necessary.

In languages such as Japanese and Thai, which also lack explicit word separators, our approach based on character-level representation and positional information may still be applicable. The core idea of incorporating external domain knowledge to enhance contextual understanding is not language dependent and can be extended to other medical languages if domain-specific resources are available.

For alphabetic languages like English, Spanish, or French, where token boundaries are more clearly defined and the writing system differs substantially, some components of the current model may require adjustment. The importance of character-level features may decrease, while word-level or phrase-level embeddings may play a more dominant role. Nevertheless, the integration of structured medical knowledge remains valuable for improving recognition of rare or ambiguous terms.

A major challenge in cross-lingual adaptation lies in the availability of appropriate resources. English has well-established clinical corpora, ontologies, and pre-trained models such as BioBERT and ClinicalBERT. In contrast, many other languages do not yet have comparable resources, which makes adaptation more difficult. However, recent progress in multilingual pre-trained models such as multilingual BERT, XLM RoBERTa, and biomedical versions of XLM suggests that extending our framework to other languages is becoming increasingly feasible.

Future work will explore these directions by incorporating multilingual encoders and evaluating the model on non-Chinese clinical datasets when available. This would help assess the generalization capacity of our method and broaden its potential use in international clinical applications.

## 5 Conclusion

This paper proposes a named entity recognition method for Chinese electronic medical records that integrates medical knowledge graph and ClinicalBERT pre-trained model. The improvement of this method is to use the ClinicalBERT model pre-trained with medical text for feature extraction of Chinese electronic medical records, and at the same time, the medical knowledge graph is embedded in the training process of the model by using the TransE method, which can effectively improve the model’s comprehension of medical terminology and semantic relationships. As shown by the experiments, the model proposed in this paper has been improved in terms of recognition accuracy and robustness.

Although the model achieves promising results on public datasets such as CCKS2019 and CCKS2020, its generalizability to real-world clinical settings remains to be further validated. In practical applications, models trained solely on public corpora may overfit to specific annotation styles or institution-specific patterns. To address this limitation, future work will explore domain adaptation techniques to transfer the model to EMRs from different hospitals or clinical departments. Additionally, external dataset validation using independently collected EMRs will be considered to assess the robustness and adaptability of the proposed method in diverse clinical environments.

In addition, the TransE knowledge graph embedding method used in this experiment relies more on negative sampling, generating negative samples is the key to the training effect, and simple on-the-fly replacement may generate low-quality negative samples, thus imaging the performance of the model. Therefore, in future work, the embedding of the knowledge graph can be further optimised, such as using semantic matching-based or neural network-based embedding models, to improve the model’s ability to model complex semantics as well as to promote the model to make full use of the local or global structural information of the knowledge graph to generate high-quality entity and relationship embeddings, so as to further improve the recognition effect of the model.

## Data Availability

The datasets used in this study are sourced from the CCKS2019 and CCKS2020 shared tasks on medical named entity recognition, organized by the Chinese Information Processing Society of China (CIPS). Both datasets consist of de-identified Chinese electronic medical records annotated with multiple categories of medical entities, including diseases, symptoms, drugs, examinations, treatments, and anatomical parts. Access to these datasets requires official registration through the corresponding CCKS competition platforms and is restricted to non-commercial academic research purposes. The datasets are not publicly available for direct download due to privacy and ethical considerations, but can be obtained upon request from the organizers.
