# Supplementary material for: Named entity recognition for Chinese electronic medical records by integrating knowledge graph and ClinicalBERT
Source: Front Artif Intell. 2025 Sep 10;8:1634774. doi: 10.3389/frai.2025.1634774 (PMC12457354; doi:10.3389/frai.2025.1634774)
Supplement: Supplementary file 1 [file Supplementary_Material.DOCX]

Supplementary Material

# Supplementary Data

**Code of building knowledge graph**

import os

import json

import random

from py2neo import Graph, Node

class MedicalGraph:

def __init__(self):

cur_dir = '/'.join(os.path.abspath(__file__).split('/')[:-1])

self.data_path = os.path.join(cur_dir, 'G:/new2019/data/medical.json')

self.g = Graph("bolt://localhost:7687", user="neo4j", password="08170914")

def read_nodes(self, limit=None, random_selection=False):

drugs, checks, departments, producers, diseases, symptoms = [], [], [], [], [], []

disease_infos = []

rels_department, rels_commonddrug, rels_recommanddrug = [], [], []

rels_check, rels_drug_producer, rels_symptom, rels_acompany, rels_category = [], [], [], [], []

count = 0

data_list = list(open(self.data_path, encoding='utf-8'))

if random_selection:

data_list = random.sample(data_list, limit)

else:

data_list = data_list[:limit]

for data in data_list:

disease_dict = {}

count += 1

print(count)

data_json = json.loads(data)

disease = data_json['name']

disease_dict['name'] = disease

diseases.append(disease)

disease_dict['desc'] = ''

disease_dict['prevent'] = ''

disease_dict['cause'] = ''

disease_dict['easy_get'] = ''

disease_dict['cure_department'] = ''

disease_dict['cure_way'] = ''

disease_dict['cure_lasttime'] = ''

disease_dict['symptom'] = ''

disease_dict['cured_prob'] = ''

if 'symptom' in data_json:

symptoms += data_json['symptom']

for symptom in data_json['symptom']:

rels_symptom.append([disease, symptom])

if 'acompany' in data_json:

for acompany in data_json['acompany']:

rels_acompany.append([disease, acompany])

if 'desc' in data_json:

disease_dict['desc'] = data_json['desc']

if 'prevent' in data_json:

disease_dict['prevent'] = data_json['prevent']

if 'cause' in data_json:

disease_dict['cause'] = data_json['cause']

if 'get_prob' in data_json:

disease_dict['get_prob'] = data_json['get_prob']

if 'easy_get' in data_json:

disease_dict['easy_get'] = data_json['easy_get']

if 'cure_department' in data_json:

cure_department = data_json['cure_department']

if len(cure_department) == 1:

rels_category.append([disease, cure_department[0]])

if len(cure_department) == 2:

big, small = cure_department[0], cure_department[1]

rels_department.append([small, big])

rels_category.append([disease, small])

disease_dict['cure_department'] = cure_department

departments += cure_department

if 'cure_way' in data_json:

disease_dict['cure_way'] = data_json['cure_way']

if 'cure_lasttime' in data_json:

disease_dict['cure_lasttime'] = data_json['cure_lasttime']

if 'cured_prob' in data_json:

disease_dict['cured_prob'] = data_json['cured_prob']

if 'common_drug' in data_json:

common_drug = data_json['common_drug']

for drug in common_drug:

rels_commonddrug.append([disease, drug])

drugs += common_drug

if 'recommand_drug' in data_json:

recommand_drug = data_json['recommand_drug']

drugs += recommand_drug

for drug in recommand_drug:

rels_recommanddrug.append([disease, drug])

if 'check' in data_json:

check = data_json['check']

for _check in check:

rels_check.append([disease, _check])

checks += check

if 'drug_detail' in data_json:

drug_detail = data_json['drug_detail']

producer = [i.split('(')[0] for i in drug_detail]

rels_drug_producer += [[i.split('(')[0], i.split('(')[-1].replace(')', '')] for i in drug_detail]

producers += producer

disease_infos.append(disease_dict)

return set(drugs), set(checks), set(departments), set(producers), set(symptoms), set(diseases), disease_infos, \

rels_check, rels_department, rels_commonddrug, rels_drug_producer, rels_recommanddrug, \

rels_symptom, rels_acompany, rels_category

def create_node(self, label, nodes):

count = 0

for node_name in nodes:

node = Node(label, name=node_name)

self.g.create(node)

count += 1

print(count, len(nodes))

return

def create_diseases_nodes(self, disease_infos):

count = 0

for disease_dict in disease_infos:

node = Node("Disease", name=disease_dict['name'], desc=disease_dict['desc'],

prevent=disease_dict['prevent'], cause=disease_dict['cause'],

easy_get=disease_dict['easy_get'], cure_lasttime=disease_dict['cure_lasttime'],

cure_department=disease_dict['cure_department'], cure_way=disease_dict['cure_way'],

cured_prob=disease_dict['cured_prob'])

self.g.create(node)

count += 1

print(count)

return

def create_graphnodes(self, limit=20, random_selection=False):

Drugs, Checks, Departments, Producers, Symptoms, Diseases, disease_infos, rels_check, rels_department, rels_commonddrug, rels_drug_producer, rels_recommanddrug, rels_symptom, rels_acompany, rels_category = self.read_nodes(limit, random_selection)

self.create_diseases_nodes(disease_infos)

self.create_node('Drug', Drugs)

print(len(Drugs))

self.create_node('Check', Checks)

print(len(Checks))

self.create_node('Department', Departments)

print(len(Departments))

self.create_node('Producer', Producers)

print(len(Producers))

self.create_node('Symptom', Symptoms)

return

def create_graphrels(self):

Drugs, Checks, Departments, Producers, Symptoms, Diseases, disease_infos, rels_check, rels_department, rels_commonddrug, rels_drug_producer, rels_recommanddrug, rels_symptom, rels_acompany, rels_category = self.read_nodes()

self.create_relationship('Department', 'Department', rels_department, 'belongs_to', '属于')

self.create_relationship('Disease', 'Drug', rels_commonddrug, 'common_drug', '常用药品')

self.create_relationship('Producer', 'Drug', rels_drug_producer, 'drugs_of', '生产药品')

self.create_relationship('Disease', 'Drug', rels_recommanddrug, 'recommand_drug', '好评药品')

self.create_relationship('Disease', 'Check', rels_check, 'need_check', '诊断检查')

self.create_relationship('Disease', 'Symptom', rels_symptom, 'has_symptom', '症状')

self.create_relationship('Disease', 'Disease', rels_acompany, 'acompany_with', '并发症')

self.create_relationship('Disease', 'Department', rels_category, 'belongs_to', '所属科室')

def create_relationship(self, start_node, end_node, edges, rel_type, rel_name):

count = 0

set_edges = []

for edge in edges:

set_edges.append('###'.join(edge))

all = len(set(set_edges))

for edge in set(set_edges):

p, q = edge.split('###')

query = "match(p:%s),(q:%s) where p.name='%s' and q.name='%s' create (p)-[rel:%s{name:'%s'}]->(q)" % (

start_node, end_node, p, q, rel_type, rel_name)

try:

self.g.run(query)

count += 1

print(rel_type, count, all)

except Exception as e:

print(e)

return

def export_data(self):

Drugs, Checks, Departments, Producers, Symptoms, Diseases, disease_infos, rels_check, rels_department, rels_commonddrug, rels_drug_producer, rels_recommanddrug, rels_symptom, rels_acompany, rels_category = self.read_nodes()

with open('drug.txt', 'w+') as f_drug, open('check.txt', 'w+') as f_check, \

open('department.txt', 'w+') as f_department, open('producer.txt', 'w+') as f_producer, \

open('symptoms.txt', 'w+') as f_symptom, open('disease.txt', 'w+') as f_disease:

f_drug.write('\n'.join(list(Drugs)))

f_check.write('\n'.join(list(Checks)))

f_department.write('\n'.join(list(Departments)))

f_producer.write('\n'.join(list(Producers)))

f_symptom.write('\n'.join(list(Symptoms)))

f_disease.write('\n'.join(list(Diseases)))

return

if __name__ == '__main__':

handler = MedicalGraph()

print("step1:导入图谱节点中")

handler.create_graphnodes(limit=100, random_selection=True)

print("step2:导入图谱边中")

handler.create_graphrels()
